# A Phase 1 Study To Assess the Pharmacokinetics of Intravenous Plazomicin in Adult Subjects with Varying Degrees of Renal Function

**DOI:** 10.1128/AAC.01128-18

**Published:** 2018-11-26

**Authors:** Allison S. Komirenko, Valerie Riddle, Jacqueline A. Gibbons, Scott Van Wart, Julie D. Seroogy

**Affiliations:** a Achaogen, Inc., South San Francisco, California, USA; b BioPharmAdvisors LLC, Parrish, Florida, USA; c Institute for Clinical Pharmacodynamics, Schenectady, New York, USA

**Keywords:** aminoglycosides, antimicrobial agents, pharmacokinetics, renal impairment

## Abstract

Plazomicin is an FDA-approved aminoglycoside for the treatment of complicated urinary tract infections. In this open-label study, 24 adults with normal renal function or mild, moderate, or severe renal impairment (*n* = 6 per group) received a single 7.5-mg/kg of body weight dose of plazomicin as a 30-min intravenous infusion. Total clearance declined with renal impairment, resulting in 1.98-fold and 4.42-fold higher plazomicin exposures, as measured by the area under the concentration-time curve from 0 h to infinity, in subjects with moderate and severe impairment, respectively, than in subjects with normal renal function. (This study has been registered at ClinicalTrials.gov under identifier NCT01462136.)

## Abstract (precis)

Plazomicin is an FDA-approved aminoglycoside for the treatment of complicated urinary tract infections. In this open-label study, 24 adults with normal renal function or mild, moderate, or severe renal impairment (*n* = 6 per group) received a single 7.5-mg/kg of body weight dose of plazomicin as a 30-min intravenous infusion.

## INTRODUCTION

Gram-negative bacteria have become increasingly resistant to multiple antibiotics (1–3), and treatment options for infections due to multidrug-resistant Enterobacteriaceae are limited (4, 5). Plazomicin is an aminoglycoside that was engineered to overcome aminoglycoside-modifying enzymes, the most common mechanism of aminoglycoside resistance in Enterobacteriaceae. *In vitro*, plazomicin has bactericidal activity against multidrug-resistant Enterobacteriaceae, including strains that produce extended-spectrum β-lactamases (6), carbapenemases (7, 8), and most aminoglycoside-modifying enzymes (9, 10). Plazomicin is approved by the U.S. Food and Drug Administration for the treatment of complicated urinary tract infections, including pyelonephritis (11).

Similar to other aminoglycosides, plazomicin displays linear dose-proportional pharmacokinetics (PK) (12, 13), has low plasma protein binding (∼20%), and does not undergo metabolism. As plazomicin is eliminated primarily via urinary excretion of the parent drug (14), we evaluated the impact of renal function on the PK of plazomicin.

This open-label phase 1 study (ClinicalTrials registration no. NCT01462136) was conducted at three U.S. centers from 2011 to 2012. The protocol was approved by the institutional review board at each site. All subjects provided written informed consent. The study included a screening period of ≤21 days, treatment on day 1, collection of PK samples on days 1 to 5, and follow-up evaluation on day 14. Adults with normal renal function or preexisting renal impairment age 18 to 75 years with a body mass index (BMI) of 19 to 32 kg/m^2^ and body weight of ≥40 kg were eligible for enrollment. Subjects requiring hemodialysis or peritoneal dialysis were excluded. Eligible subjects were stratified based on the mean of two predose creatinine clearance (CL_CR_) values, as estimated by the Cockcroft and Gault equation (15), and were enrolled concurrently into the mild (CL_CR_, 60 to 89 ml/min), moderate (CL_CR_, 30 to 59 ml/min), or severe (CL_CR_, 15 to 29 ml/min) renal impairment group (*n* = 6 per group). Subjects in the normal renal function group (CL_CR_, ≥90 ml/min) were enrolled and treated only after dosing had been completed in the renal impairment groups. Staggered enrollment was employed to approximately match the demographic characteristics of subjects in the normal renal function group with those of subjects in the renal impairment groups.

A single 7.5-mg/kg of body weight dose of plazomicin was administered via a 30-min intravenous infusion. The single 7.5-mg/kg dose was selected to avoid high plazomicin exposures in subjects with severe renal impairment. Blood samples were collected before dosing and at 36 and 45 min and 1, 1.5, 3, 6, 10, 16, 24, 36, 48, 72, and 96 h after the start of infusion. Noncompartmental PK analysis was conducted using Phoenix WinNonlin v.6.1 (Pharsight Corp., Mountain View, CA, USA) (details are described in the supplemental material).

Twenty-four subjects were enrolled and completed the study. The mean age was 63.9 years (range, 50 to 75 years). While the renal function groups were well matched for age and sex, values for mean body weight and BMI were slightly higher in the normal renal function group than in the impaired renal function groups (Table 1).

**TABLE 1:** Subject demographic and baseline characteristics^*a*^

Characteristic	Normal renal function (*n* = 6)	Mild renal impairment (*n* = 6)	Moderate renal impairment (*n* = 6)	Severe renal impairment (*n* = 6)	Total (*N* = 24)
Age (yr)	61.8 ± 8.08	62.7 ± 7.55	65.7 ± 9.27	65.3 ± 7.28	63.9 ± 7.73
Female (no. [%])	3 (50.0)	2 (33.3)	3 (50.0)	4 (66.7)	12 (50.0)
White (no. [%])	5 (83.3)	5 (83.3)	5 (83.3)	4 (66.7)	19 (79.2)
Wt (mean ± SD) (kg)	83.1 ± 14.7	72.4 ± 8.5	77.7 ± 11.1	74.1 ± 21.5	76.8 ± 14.4
BMI (mean ± SD) (kg/m^2^)	28.1 ± 3.93	25.7 ± 2.41	27.4 ± 2.40	27.4 ± 3.89	27.2 ± 3.15
CL_CR_ (mean ± SD) (ml/min)	115 ± 18.5	75.9 ± 5.02	49.5 ± 8.05	21.2 ± 6.95	65.5 ± 36.9

a BMI, body mass index; CL_CR_, creatinine clearance; Wt, weight.

**FIG 1:**
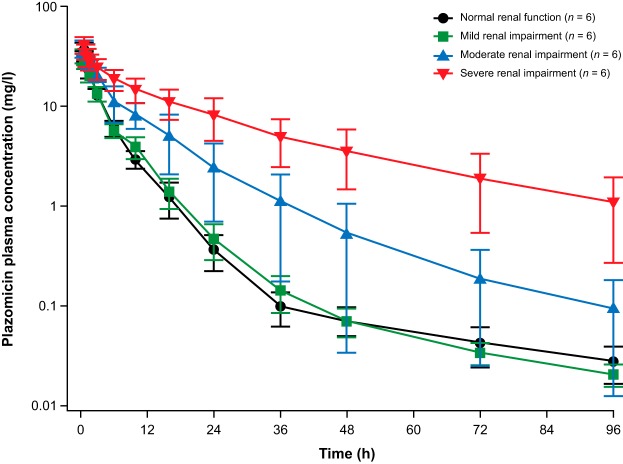
Semilog plot of the mean (SD) plazomicin plasma concentration-time profile by renal function group.

Plazomicin plasma drug concentrations declined in a multiphasic manner over time in each renal function group (Fig. 1). Subjects with mild renal impairment had plazomicin plasma concentration-time profiles that were comparable to those in subjects with normal renal function. Mean plazomicin plasma concentrations were higher in subjects with moderate and severe renal impairment than in subjects with normal renal function.

Following a single dose of plazomicin, maximum concentration of drug in plasma (C_max_) values did not appear to be impacted by renal impairment (*P* > 0.05; Table 2). Plasma exposures based on the area under the plasma concentration-time curve from time zero to infinity (AUC_0–∞_) were similar in subjects with mild renal impairment and normal renal function, but they were 1.98-fold and 4.42-fold higher in subjects with moderate and severe impairment, respectively, than in subjects with normal renal function (Table 3). The volume of distribution at steady state (V_ss_) was approximately 20% to 30% lower in subjects with renal impairment than in subjects with normal renal function. Total clearance (CL_T_) values were not notably different in subjects with mild renal impairment versus those with normal function but were substantially lower with moderate and severe impairment than with normal renal function (Table 2).

**TABLE 2:** Plazomicin plasma pharmacokinetic parameters by renal function group^*a*^

PK parameter	Normal renal function (*n* = 6)	Mild renal impairment (*n* = 6)	Moderate renal impairment (*n* = 6)	Severe renal impairment (*n* = 6)
C_max_ (mg/liter)	37.9 ± 5.01	32.8 ± 4.30	39.2 ± 6.43	41.4 ± 7.83
AUC_0–∞_ (mg · h/liter)	136 ± 17.2	138 ± 23.7	281 ± 96.0	647 ± 259
V_ss_ (liters)	36.0 ± 7.76	28.5 ± 2.17	25.8 ± 6.96	25.1 ± 7.89
CL_T_ (liters/h)	4.64 ± 1.17	3.98 ± 0.481	2.25 ± 0.685	0.96 ± 0.379

a All values are reported as mean ± SD. PK, pharmacokinetics; C_max_, maximum concentration of drug in plasma; AUC_0–∞_, area under the concentration-time curve from 0 h to infinity; V_ss_, volume of distribution at steady state; CL_T_, total clearance.

**TABLE 3:** Pairwise comparisons of plazomicin plasma pharmacokinetic parameters by renal function group^*a*^

PK parameter	Pairwise group comparison geometric mean ratio (90% CI), *P* value		
	Mild renal impairment/normal renal function	Moderate renal impairment/normal renal function	Severe renal impairment/normal renal function
C_max_ (mg/liter)	0.87 (0.753–0.997), 0.093	1.03 (0.883–1.21), 0.719	1.08 (0.899–1.30), 0.460
AUC_0–∞_ (mg · h/liter)	1.01 (0.849–1.19), 0.942	1.98 (1.51–2.60), 0.0023	4.42 (3.04–6.44), 0.0003

a PK, pharmacokinetic; CI, confidence interval; C_max_, maximum concentration of drug in plasma; AUC_0–∞_, area under the concentration-time curve from 0 h to infinity.

A single 7.5-mg/kg dose of plazomicin was well tolerated across renal function groups. Three adverse events were reported, none of which was considered by the investigator to be related to plazomicin. The results of the safety analyses are provided in the supplemental material.

Our results show that similar to other aminoglycosides (16, 17), the PK of plazomicin is impacted by renal function. Total clearance declined with renal impairment, resulting in significantly increased plazomicin exposures with moderate and severe impairment. Given the magnitude of increases in AUC_0–∞_, adjustments to plazomicin dosing are indicated for patients with moderate or severe renal impairment to attain a range of exposures similar to those of patients with normal renal function (11).

Similar to other plazomicin PK studies and to other aminoglycosides, the V_ss_ for plazomicin approximated the extracellular fluid volume of 15 to 25 liters (12, 13, 18–20). Lower mean V_ss_ values observed with renal impairment than with normal function may in part be related to the slightly higher body weights in subjects with normal renal function than in those with renal impairment.

A strength of this study is that PK was evaluated in subjects with a broad range of baseline CL_CR_, including three subjects with CL_CR_ of <20 ml/min. An important limitation is the exclusion of subjects with end-stage renal disease requiring dialysis, precluding evaluation of the impact of renal replacement therapy on plazomicin PK. In conclusion, while dosage regimen adjustments of plazomicin do not appear to be warranted for patients with mild renal impairment, dosage adjustments are recommended in patients with moderate or severe renal impairment.

### Data availability.

Individual participant data that underlie the results reported in this article, after deidentification, will be made available (unless prohibited by applicable law) to researchers for noncommercial purposes beginning 6 months following article publication; proposals should be directed to datarequests@achaogen.com.

## Supplementary Material

Supplemental file 1
