# Supplementary material for: A Phase 1 Study To Assess the Pharmacokinetics of Intravenous Plazomicin in Adult Subjects with Varying Degrees of Renal Function
Source: Antimicrob Agents Chemother. 2018 Nov 26;62(12):e01128-18. doi: 10.1128/AAC.01128-18 (PMC6256775; doi:10.1128/AAC.01128-18)
Supplement: Supplemental file 1 [file zac012187667s1.pdf]

SUPPLEMENTAL MATERIAL

A Phase 1 Study to Assess the Pharmacokinetics of Intravenous Plazomicin in Adult Subjects with Varying Degrees of Renal Function

RESULTS

Safety and tolerability. Of the 24 subjects enrolled, three subjects (one each with normal renal function, mild renal impairment, and severe renal impairment) experienced an adverse event (AE), consisting of a mild upper respiratory tract infection, a mild headache, and moderate epicondylitis, respectively. None of these AEs was considered by the investigator to be related to plazomicin. No serious or severe AEs or deaths were reported during the study. Laboratory test results, vital signs, and electrocardiogram results were generally stable without any clinically significant changes.

Aminoglycoside class–associated toxicities of nephrotoxicity and ototoxicity were specifically monitored in this study. In general, serum creatinine measurements remained stable throughout the study, with minimal changes from baseline values. In subjects with normal renal function, mild renal impairment, or moderate renal impairment, no clinically meaningful (≥0.5 mg/dl) increases in serum creatinine (1) were observed relative to baseline. Of six subjects in the severe renal impairment group, an increase in serum creatinine ≥0.5 mg/dl above baseline was observed for two subjects with a baseline creatinine clearance (CL_{CR}) of 18.0 ml/min and 10.1 ml/min. The lowest CL_{CR} levels observed for these subjects were 16.0 ml/min and 9.0 ml/min, respectively (Table S1). As these changes were observed at the last study visit (13 days postdose), additional serum creatinine measurements were not collected.

Cochlear and vestibular function were monitored by pure tone audiometry (PTA) with bone conduction and the modified Romberg test, respectively. Audiometry was introduced with a protocol amendment, at which point 10 subjects had already completed the study. Of the 14 subjects evaluated by PTA with bone conduction, none experienced a clinically significant change in hearing from baseline at the follow-up visits 4 and 13 days postdose based on the prespecified criteria for clinical significance. Results of modified Romberg testing showed that no subject demonstrated a decline in test results following dosing. Overall, the results of both cochlear and vestibular function testing were not indicative of ototoxicity.

MATERIALS AND METHODS

Inclusion criteria. Subjects had normal renal function or preexisting renal impairment and were required to have a stable disease process responsible for any underlying renal dysfunction at the time of study entry and no acute renal disease in the year before dosing.

Exclusion criteria. Subjects were excluded if they required hemodialysis or peritoneal dialysis, took medications known to cause changes in renal function in the 2 weeks before dosing (e.g., nonsteroidal anti-inflammatory agents), had a history of significant hearing loss or family history of hearing loss (excluding age-related hearing loss), had a prior diagnosis of Ménière’s disease or sensorineural hearing loss, or had myasthenia gravis or any other neuromuscular disorder. Subjects with end-stage renal disease (corresponding to a mean predose CL_{CR} <15 ml/min) and not on dialysis were permitted upon approval from the investigator and the medical monitor.

Safety and tolerability. Safety and tolerability were monitored through AEs, vital signs, laboratory tests, physical examinations, electrocardiograms, PTA (up to 10,000 Hz) with bone conduction (up to 4,000 Hz), and modified Romberg testing. Clinical significance of postdose PTA results was determined based on prespecified criteria, which included a negative shift of ≥10 dB at two consecutive frequencies, with further shifts (at subsequent test points) of ≥10 dB at three subsequent consecutive frequencies. The modified Romberg test evaluates neurological function, including proprioception, vestibular function, and vision (2). A *post hoc* analysis was conducted to analyze the proportion of subjects for whom a serum creatinine increase ≥0.5 mg/dl above baseline was observed.

Bioanalytical methods. K_2EDTA plasma samples were analyzed for plazomicin concentrations using a validated high-performance liquid chromatography/tandem mass spectrometry (HPLC/MS/MS) method at Alturas Analytics, Inc. (Moscow, ID, USA) with a lower limit of quantification of 0.01 mg/liter. Plasma samples were extracted with 20% trichloroacetic acid after adding stable labeled internal standard. An aliquot of the extract was injected onto an API4000 HPLC/MS/MS system (column: Supelco Discovery HS C18, 2.1 × 50 mm, 3 μm; mobile phases: 0.05% heptafluorobutyric acid [HFBA] in water and 0.05% HFBA in acetonitrile). The overall precision of the quality controls and standards was within 10.6% coefficient of variation (CV) and the mean accuracy ranged from 95.7 to 103%.

Pharmacokinetic analysis. Maximum plasma concentration (C_{max}) was determined by direct observation. The area under the plasma concentration-time curve from time zero to the last quantifiable sample ($AUC_{0–t}$) was calculated using the linear trapezoidal

rule. A linear regression was performed on the log-linear terminal portion of the plasma concentration-time curve to calculate the apparent terminal-phase elimination rate constant (λ_z). Area under the plasma concentration-time curve from time zero to infinity ($AUC_{0-\infty}$) was determined by summing the AUC_{0-t} with the extrapolated area from the time of the last quantifiable sample (t_{last}) to infinity, as estimated by C_t/λ_z, where C_t is the last measurable concentration. Mean residence time was calculated as the ratio of $AUMC_{0-\infty}$ to $AUC_{0-\infty}$ minus the mean absorption time, which is equal to 15 min, or one-half of the infusion duration time. Steady-state volume of distribution (V_{ss}) was determined as the product of total clearance (CL_T) and mean residence time. CL_T was calculated as the plazomicin dose divided by $AUC_{0-\infty}$.

Statistical analysis. Summary statistics for plazomicin PK parameters were calculated for each renal function group, and multiple pairwise comparisons were conducted to compare the geometric means for each PK parameter. Two-sample *t*-tests were performed in SAS version 9.2 (SAS Institute Inc., Cary, NC, USA). A test for equal variances was first performed using an *F*-test, and if statistically significant ($\alpha = 0.05$), the unequal variances (Satterthwaite) *P*-value was reported instead of the pooled equal variances *P*-value. Pairwise comparisons of the geometric mean ratios were tested for statistical significance ($\alpha = 0.05$) and also analyzed to determine if the lower or upper bound of the 90% confidence intervals around the geometric mean ratio excluded 1.

REFERENCES

1. Waikar SS, Bonventre JV. 2009. Creatinine kinetics and the definition of acute kidney injury. J Am Soc Nephrol 20:672-679.

2. Notermans NC, van Dijk GW, van der Graaf Y, van Gijn J, Wokke JH. 1994. Measuring ataxia: quantification based on the standard neurological examination. J Neurol Neurosurg Psychiatry 57:22-26.

TABLE S1 CL_{CR} and serum creatinine levels for subjects with any serum creatinine increase ≥0.5 mg/dl above baseline

	**Baseline CL_{CR} (ml/min)**	**Lowest CL_{CR} (ml/min)**	**Baseline serum creatinine (mg/dl)**	**Maximum serum creatinine (mg/dl)**	**Absolute change in serum creatinine (mg/dl)**	**Fold-increase in serum creatinine from baseline**
Subject 1	18.0	16.0	8.11	8.80[a]	0.69	1.1
Subject 2	10.1	9.0	5.30	6.20[a]	0.90	1.2

[a]Values were observed at 13 days postdose.
